# Supplementary material for: A dataset of insect sounds from 459 species for bioacoustic machine learning
Source: Sci Data. 2026 Mar 27;13:499. doi: 10.1038/s41597-026-07123-4 (PMC13035933; doi:10.1038/s41597-026-07123-4)
Supplement: Supplementary file 1 — Supplementary information: Classifier performance [file 41597_2026_7123_MOESM1_ESM.pdf]

Supplementary information: Classifier performance

InsectSet66:

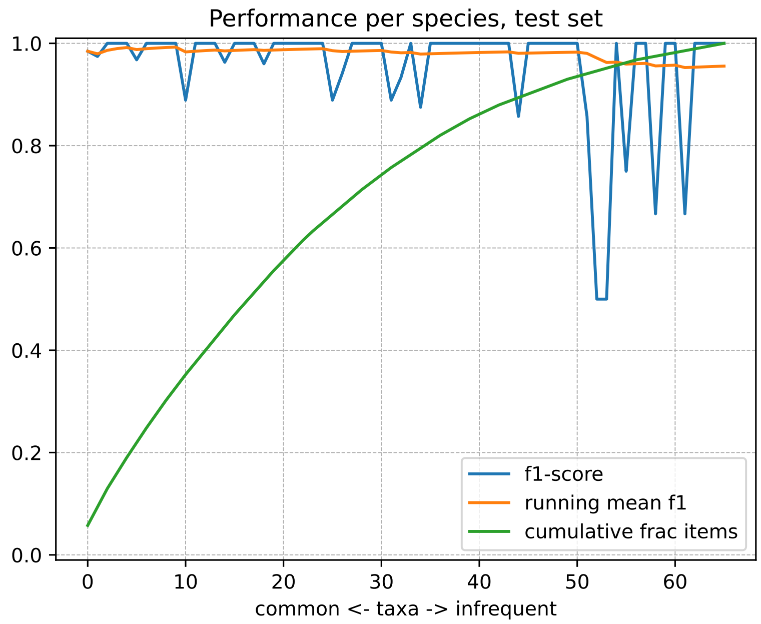

Figure 1: Per-species classification performance of InsectEffNet trained on InsectSet66, evaluated on the test set. The species are ordered on the x-axis from most common to least common in the dataset, and for each one we plot a running mean of the F1 score—meaning the mean of the F1 score for all species that are equally or more common.

3 InsectSet459:

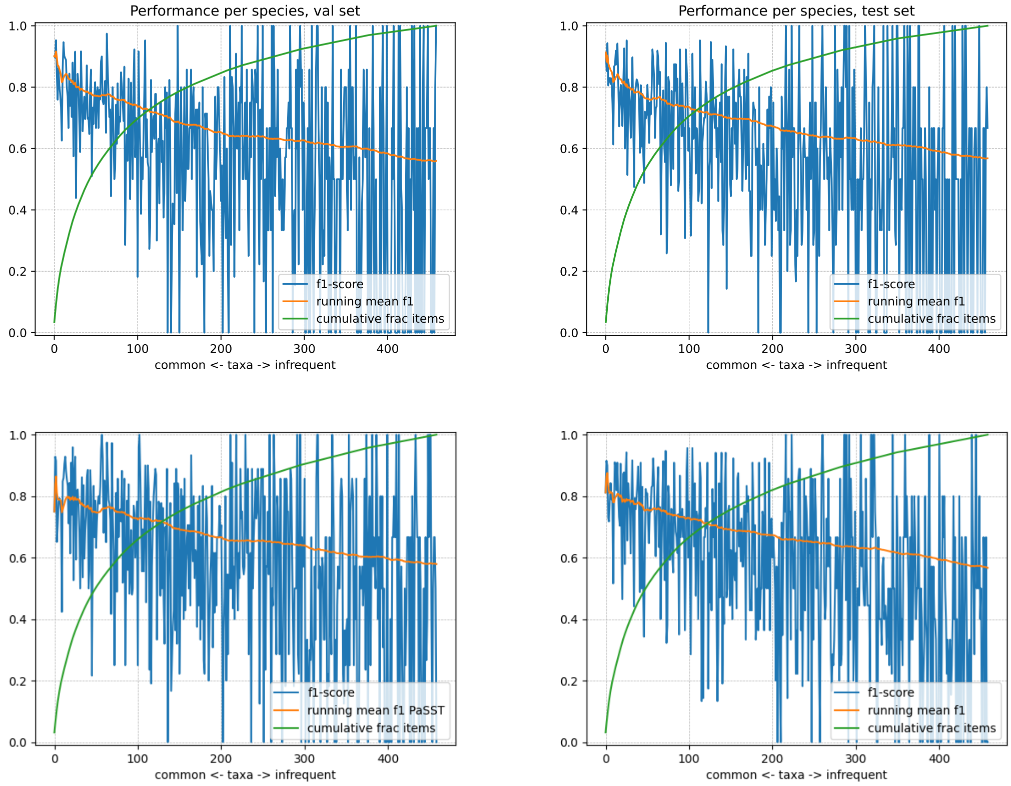

Figure 2: Per-species classification performance of InsectEffNet and PaSST trained on InsectSet459, evaluated on the validation and test sets. The species are ordered on the x-axis from most common to least common in the dataset, and for each one we plot the F1-score and a running mean. a) Top left: Performance of InsectEffNet on the validation set. b) Top right: Performance of InsectEffNet on the test set. c) Bottom left: Performance of PaSST on the validation set. b) Bottom right: Performance of PaSST on the test set.
